# ‘One Size Doesn’t Fit for All’: There Is a Need for Targeted Personalized Therapy in Obstructive Sleep Apnea Syndrome

**DOI:** 10.3390/jcm11133595

**Published:** 2022-06-22

**Authors:** Athanasia Pataka

**Affiliations:** Respiratory Failure Unit, G. Papanikolaou Hospital, Aristotle University of Thessaloniki, 54124 Thessaloniki, Greece; patakath@yahoo.gr or patakath@auth.gr

The estimated prevalence of moderate to severe obstructive sleep apnea syndrome (OSA) has increased by 14–55% over the last few decades [1]. Continuous positive airway pressure (CPAP) has become the current gold standard of OSA treatment since the early 1980s [2]. CPAP has been proven to be highly effective in decreasing respiratory events during sleep and in improving the clinical manifestations of the disease [3,4,5]. However, almost 50% of the patients who are prescribed CPAP are not adherent to it at all or use it <4 h per night [6]. As a result, there is a definite need for novel treatments and for the development of new effective strategies in order to accurately predict the optimal solutions for each patient.

Diagnosis and severity of OSA have been traditionally based on the apnea-hypopnea index (AHI). However, the AHI centered approach of OSA does not seem to be sufficient nowadays in order to assess the heterogeneity of the disease and to evaluate different pathogenetic mechanisms, clinical presentations, risk factors, and response to treatment [7,8]. The high number of AHI (>30 event/h) has been related with increased morbidity and mortality. On the other hand, this not so evident for OSA patients with less frequent respiratory events and data supporting the effect of treatment with CPAP in these patients concerning the improvement of survival and cardiovascular risk are limited [9,10,11]. The selection of OSA treatment is closely related to patient outcomes and the limited adherence to CPAP leads to the search of other alternative non-CPAP treatments [6,12]. 

The heterogeneity of the pathogenesis of OSA is still poorly defined. Until recently, the most important variable for the pathogenesis of the disease was considered to be the impaired upper airway anatomy. On the other hand, other non-anatomical phenotypes, as unstable respiratory control, impaired upper airway muscle function during sleep, and a low respiratory arousal threshold may also play a significant role [13,14]. However, most of the treatment options for OSA, such as CPAP, mandibular advancement devices, upper airway surgery, and hypoglossal nerve stimulation, are focused on improving the impaired upper airway anatomy but not the other pathogenetic traits of the disease. Different interventions according to the different endotypes of each patient should be used. Upper airway surgery (e.g., palatal surgery) may be useful for those patients with problems in the anatomy of velopharynx, whereas acetazolamide or oxygen in patients with high loop gain (unstable ventilatory control) [15]. Recently, studies using different approaches, as cluster analysis, have tried to examine OSA heterogeneity aiming to identify different phenotypes in order to achieve a more personalized approach of management [16].

As there is a lack of high scientific evidence for the effective management of patients suffering from OSA who are not adherent or refuse CPAP, the European Respiratory Society (ERS) has recently updated an older statement on non-CPAP treatment [17] as new methods as hypoglossal nerve stimulation (HNS) [18] have been introduced in the last years and others, such as positional therapy, mandibular advancement devices [19], myofunctional therapy, maxillo-mandibular osteotomy, and some pharmacological agents have been reconsidered [20]. The therapeutic approach of OSA should be multidimensional also including patients’ education and help for weight reduction [21]. 

The future treatment options of OSA should be based on the unique pathophysiological traits of each patient and on the personalized selection therapy [15,22]. This may lead to combinations of different therapies as CPAP, mandibular advancement devices, and positional therapies [23,24] for the identification of the most effective and suitable treatment for each patient individually. Additionally, new pharmaceutical agents that may affect the different pathophysiological mechanisms of the disease, such as respiratory drive, arousals, and upper airway muscles [25,26,27], have been recently investigated, as well as others agents that may be used for the treatment of residual sleepiness in OSA patients [28,29,30]. 

The key goals of the future treatment of OSA should be to provide alternative efficacious management pathways beyond the traditional “one size fits all” CPAP focused approach that seems to be inefficient to many of our patients. Identification of different clinical phenotypes and their underlying pathophysiological endotypes may provide novel strategies to the treatment of the disease based on precision medicine and, as a result, further improve treatment adherence and success with CPAP (but also non-CPAP therapies) [31]. A future priority should be to integrate this ‘personalized’ approach and implement it into everyday clinical practice. A major challenge of this approach will be to update tools in pragmatic trials in order to evaluate endotyping in the real clinical practice highlighting the heterogeneity of OSA with its different outcomes in patients’ lives. 
